# Profile of Tau-Associated Selected MicroRNAs in Hospitalized COVID-19 Patients: An Exploratory Single-Center Study

**DOI:** 10.3390/cells15060503

**Published:** 2026-03-12

**Authors:** Elena Carbone, Maria Antonella Zingaropoli, Federica Perrone, Giuseppina Talarico, Patrizia Pasculli, Antonio Minni, Carla Petrella, Christian Barbato, Paola Piscopo

**Affiliations:** 1 Department of Neuroscience, Italian National Institute of Health, Viale Regina Elena, 299, 00161 Rome, Italy; elena.carbone@iss.it; 2 Department of Public Health and Infectious Diseases, Sapienza University of Rome, Viale del Policlinico, 155, 00185 Rome, Italy; mariaantonella.zingaropoli@uniroma1.it (M.A.Z.); patrizia.pasculli@uniroma1.it (P.P.); 3 National Center for Disease Prevention ad Heath Promotion, Italian National Institute of Health, Viale Regina Elena, 299, 00161 Rome, Italy; federica.perrone@iss.it; 4 Department of Human Neuroscience, Sapienza University of Rome, Viale dell’ Università, 30, 00185 Rome, Italy; giuseppina.talarico@uniroma1.it; 5 Department of Sense Organs DOS, Sapienza University of Rome, Viale del Policlinico, 155, 00161 Rome, Italy; antonio.minni@uniroma1.it; 6 Division of Otolaryngology-Head and Neck Surgery, ASL Rieti-Sapienza University, Ospedale San Camillo de Lellis, Viale Kennedy, 02100 Rieti, Italy; 7 Interdisciplinary Department of Well-Being, Health and Environmental Sustainability (BeSSA), Sapienza University of Rome, Via delle Fontanelle, 02100 Rieti, Italy; 8 Institute of Biochemistry and Cell Biology (IBBC), National Research Council (CNR), Sapienza University of Rome, Policlinico Umberto I, Viale del Policlinico, 155, 00161 Rome, Italy; carla.petrella@cnr.it

**Keywords:** COVID-19, circulating microRNAs, miR-92a-3p, miR-320a, miR-320b, neuroinflammation, neurodegeneration, tau pathway, plasma biomarkers

## Abstract

Tau-associated microRNAs have been implicated in neurodegenerative disorders, yet their behavior during SARS-CoV-2 infection remains insufficiently understood. The aim of this study was to quantify circulating levels of miR-92a-3p, miR-320a, and miR-320b in hospitalized COVID-19 patients and evaluate their relationship with disease severity and established biomarkers of neuroinflammation and neurodegeneration. We conducted a retrospective single-center study including 38 hospitalized COVID-19 patients and 12 healthy controls. MicroRNA plasma levels were quantified by RT-qPCR. Patients were stratified by ARDS severity and ventilation requirements. Correlations between miRNAs and previously published biomarkers were examined. All three miRNAs were elevated in COVID-19 patients compared to healthy controls. miR-92a-3p and miR-320a were increased in both severe and non-severe cases, while miR-320b was significantly elevated only in severe disease. No statistically significant correlations were observed between miRNA levels and NfL, GFAP, MMP-9, or other biomarkers in COVID-19 patients. Tau-associated circulating microRNAs appear dysregulated in acute SARS-CoV-2 infection, but their relationship to neurological injury remains unclear. These findings are preliminary and require validation in larger, longitudinal cohorts with standardized neurological outcomes.

## 1. Introduction

The global spread of SARS-CoV-2, the virus responsible for the COVID-19 pandemic, has been increasingly linked to the onset, progression, and exacerbation of neurological disorders (NDs) [1]. Recent evidence has shown that COVID-19 is not only a respiratory disease but also a systemic disorder with a significant neurological component. Several studies have reported acute and subacute neurological manifestations—including encephalopathy, cerebrovascular events, anosmia, cognitive disturbances, and autonomic dysfunction—suggesting that SARS-CoV-2 may affect both central and peripheral nervous systems through a combination of inflammatory, vascular, and immune-mediated mechanisms [2]. Older and frail individuals appear particularly vulnerable to these complications, and large national surveys, including those conducted in Italy, have highlighted the substantial clinical impact of COVID-19 in this population [3]. Persistent symptoms following infection, collectively referred to as “Long COVID,” often include cognitive dysfunction, also known as NeuroCOVID [4]. These neurological manifestations range from impaired concentration and difficulties in daily functioning to short-term memory deficits and frontal syndrome [5]. Research into biomarkers for both NeuroCOVID and NDs is needed to uncover shared molecular alterations and pathways, clarifying whether, and to what extent, SARS-CoV-2 contributes to or accelerates neurocognitive decline. Previous studies have demonstrated that elevated serum levels of neurofilament light chain (NfL) in COVID-19 patients correlate with severe or fatal outcomes [6]. Increased blood NfL has been proposed not only as a biomarker of COVID-19 severity, associated with worse clinical trajectories and prolonged hospitalization, but also as a potential indicator of NeuroCOVID [7,8]. Notably, altered NfL levels are also observed in several NDs, including Alzheimer disease (AD) and Frontotemporal dementia (FTD) [9].

Recent evidence also indicates that SARS-CoV-2 may affect tau biology. Post-mortem analyses of individuals who recovered from COVID-19 revealed abnormal accumulation of hyperphosphorylated tau in the hippocampus and entorhinal cortex, accompanied by persistent neuroinflammation, even months after infection [10]. Experimental studies further demonstrate that SARS-CoV-2 infection induces pathological tau phosphorylation, aggregation, and altered subcellular distribution in neuronal models and in K18-hACE2 mice, suggesting a direct viral impact on tau homeostasis [11]. Additionally, the SARS-CoV-2 main protease (3CLpro) was shown to cleave tau and promote aggregation, providing another mechanistic link between the virus and tau dysregulation [12]. Together, these findings support growing interest in tau-related pathways as potential contributors to acute and post-acute neurological complications of COVID-19.

Although neuropathological findings and clinical observations suggest that NeuroCOVID may increase the risk of neurodegeneration, it remains uncertain whether COVID-19 triggers or accelerates chronic neurodegenerative processes [1,13]. This underscores the urgent need for predictive biomarkers of post-pandemic neurological outcomes. Epigenetic regulatory mechanisms, particularly microRNAs (miRNAs), play a crucial role in shaping the host response to viral infections and in modulating inflammatory and neurovascular pathways. MiRNAs are small, non-coding RNAs that regulate gene expression post-transcriptionally and influence processes such as endothelial function, immune activation, oxidative stress, and neuronal homeostasis [14,15]. Several studies have described dysregulated miRNA signatures in COVID-19, with specific miRNAs correlating with disease severity, cytokine activation, and endothelial injury [16]. Given their stability in circulation and their ability to reflect alterations occurring in distant tissues, circulating miRNAs are increasingly regarded as promising minimally invasive biomarkers for monitoring COVID-19-related systemic and neurological involvement. MicroRNAs (miRNAs) have emerged as promising biomarkers for diagnosing and prognosing central nervous system disorders [17]. Highly expressed in the brain, miRNAs play critical roles in the occurrence and development of NDs. Among the miRNAs altered in viral and neuroinflammatory conditions, some—including miR-92a-3p, miR-320a, and miR-320b—have been previously associated with pathways relevant to neuronal structure, cytoskeletal regulation, and tau-related cellular processes in experimental models [18]. In the present study, these miRNAs are considered candidates of interest based on their known biological roles and on the literature linking them to processes potentially relevant to NeuroCOVID. In fact, our recent work experimentally demonstrated that miR-92a-3p, miR-320a and miR-320b directly interact with the MAPT transcript, as shown by luciferase reporter assays and gain- and loss-of-function experiments, confirming their ability to modulate tau expression [18]. These findings provide the rationale for referring to these molecules as “tau-associated miRNAs” in the present exploratory study. This pattern of dysregulation in AD and FTD [18,19] mirrors recent observations in COVID-19, where alterations in these miRNAs and evidence of Tau perturbation have been documented, suggesting convergence on shared neurodegenerative pathways [20,21]. Additionally, miR-92a-3p is involved in endothelial activation and vascular inflammation, which are key mechanisms implicated in COVID-19-related microvascular dysfunction, whereas members of the miR-320 family regulate extracellular matrix remodeling and have been linked to modulation of MMP-9, a protease associated with blood–brain barrier permeability [22]. This study aimed to investigate whether plasma levels of three tau-associated microRNAs (miR-92a-3p, miR-320a, and miR-320b) differ between COVID-19 patients and healthy controls, and to explore their association with disease severity, ventilation requirements, and clinical outcomes.

## 2. Materials and Methods

### 2.1. Study Population

A retrospective study was conducted at a single healthcare facility, involving COVID-19 patients admitted between March 2020 and March 2021 to the Public Health and Infectious Diseases Department of Umberto I Hospital, Sapienza University of Rome. Based on power analysis calculator for the sample size in a pilot study, we obtained that with at least 30 total sample size we could guarantee an actual power of 0.95 (for additional details see Supplementary Materials). The sample included only adults (over eighteen years of age) at the time of admission. As previously specified [23], pneumonia due to COVID-19 was confirmed by high-resolution lung-computed tomography scan combined with detection of SARS-CoV-2 RNA obtained from a nasopharyngeal swab. This detection was performed using a commercial reverse transcriptase polymerase chain reaction (RT-PCR) kit, following the manufacturer’s instructions (RealStar^®^ SARS-CoV-2 Altona Diagnostics). These patients were selected at the time of admission for the absence of comorbidities or other confounding factors such as hypertension, cardiovascular diseases, diabetes and without ongoing therapy. Initially, patients were divided into two categories, severe and non-severe, based on the severity of COVID-19 during the acute phase of the disease (assessed based on the onset of acute respiratory distress syndrome [ARDS]). Severe and non-severe groups were further classified into the following four subgroups based on the maximum oxygen supply/ventilation support required during hospitalization: the severe group into invasive mechanical ventilation via orotracheal intubation (IOT) and noninvasive ventilation (NIV) subgroups and the non-severe group into Venturi mask for oxygen (VMK) and room air (AA) subgroups. Additionally, a small group of hospitalized patients, referred to as the “deceased group,” died, but it was not possible to monitor them and divide them into severity subgroups. Furthermore, unlike the other recruited patients, as expected, the deceased group had previous cardiovascular diseases and diabetes, which were not evident at the time of admission. An additional stratification was performed based on the presence or absence of neurological symptoms, such as confusion, syncope headache, paresthesia brain fog, specifically, defined by a neurologist disease physician, on hospital admission. Lastly, healthy control (HC) group was enrolled matched for age and gender, with a negative nasopharyngeal swab for SARS-CoV-2 RNA detection, undetectable anti-SARS-CoV-2-specific IgG and without any symptoms.

### 2.2. Plasma Collection

The collection of plasma samples followed previously validated procedures [18]. During routine clinical testing whole blood was collected in heparin-containing tubes; samples were centrifuged at 1600× *g*, 4 °C for 15 min. The top layer, plasma, was aliquoted in 250 µL and stored at −80 °C.

### 2.3. RNA Extraction and miRNA Quantification

The miRNeasy Serum/Plasma Kit (Qiagen, Hilden, Germany) was used for miRNA extraction from plasma samples. An exogenous synthetic spike-in control (miR-39; MiRXES) was added to each sample before extraction to monitor extraction efficiency and to detect potential technical variability among samples. Multiple QC steps were implemented to ensure data reliability.

The stability and consistency of the exogenous spike-in miR-39 Ct values across samples were evaluated to confirm uniform extraction performance and RT-qPCR amplification efficiency. Samples showing aberrant miR-39 Ct values were flagged for potential exclusion; however, no sample exceeded QC thresholds. Hemolysis was evaluated using hemolysis-sensitive and hemolysis-resistant miRNAs (standard practice in plasma miRNA studies). Specifically, we quantified hemolysis-associated miRNAs [24] and verified the absence of elevated hemolysis signatures across samples. No samples showed evidence of hemolysis exceeding accepted thresholds. cDNA synthesis was performed with the MiRXES ID3EAL cDNA kit using modified stem-loop primers. Because residual heparin is known to inhibit reverse transcription and PCR amplification by interacting with polymerases and reverse transcriptase, we implemented standard mitigation procedures recommended for plasma miRNA quantification. As suggested in previous methodological studies [25,26] dilution of cDNA is an effective approach to reduce heparin-mediated inhibition without altering relative miRNA abundance. For this reason, each cDNA sample was diluted 1:50 in nuclease-free water prior to RT-qPCR, allowing optimal amplification efficiency while maintaining assay linearity. This dilution step is widely used in heparinized plasma workflows and it prevents spurious increases in Ct values or failure of amplification. The effectiveness of this procedure was confirmed by the stable Ct distribution of the exogenous spike-in (miR-39) across samples, indicating that residual heparin did not compromise extraction or amplification performance. All reactions included negative controls (NTCs). RT-qPCR was performed using ID3EAL miRNA qPCR reagents (MiRXES) on a QuantStudio 5 Real-Time PCR System (Thermo Fisher Scientific, Waltham, MA, USA). Technical triplicates were run for each miRNA; replicates with Ct > 35 or with SD > 0.5 were evaluated individually. MiR-191-5p, a well-established endogenous reference miRNA with reported stability in plasma and serum, was quantified and used as the primary normalizer for ΔCt calculations. miR-191-5p showed minimal intra- and inter-group variability and passed all stability criteria. Its stability was formally validated using NormFinder, which identified miR-191-5p as the most stable candidate among the reference miRNAs evaluated. Normalization was performed using the 2^−ΔCt method, where ΔCt = Ct (miRNA of interest) − Ct (miR-191-5p).

### 2.4. Statistical Analysis

Given the small sample size and potential non-normality of ΔCt values, we applied both parametric and non-parametric approaches. For two-group comparisons: Student’s *t*-test and Mann–Whitney U test, for multiple-group comparisons: ANOVA and Kruskal–Wallis test and Post hoc testing Benjamini–Hochberg false discovery rate. The Spearman correlation test was performed to investigate the association between miRNAs and the neuroinflammation and neurodegeneration considered in the present study. As the study was exploratory and not powered for subgroup comparisons, *p*-values in this case are interpreted descriptively.

## 3. Results

### 3.1. Study Population

As shown in Table 1, the study included 38 hospitalized COVID-19 patients (20 males, 18 females; mean age 67 ± 14 years) and 12 HC (six males, six females; mean age 60 ± 9 years). Initially, patients were classified as severe (20/38) or non-severe (18/38) based on the development of ARDS during hospitalization. The enrolled patients were then further divided into four subgroups (Table 2) according to the highest level of oxygen therapy or ventilatory support required during their stay: four required invasive mechanical ventilation (IOT), 16 non-invasive ventilation (NIV), six Venturi mask oxygen therapy (VMK), and 12 room air (AA). Six patients died during hospitalization (five males, one female; mean age 59.8 years), indicated as deceased group. The clinical characteristics of the patients have already been described elsewhere [23]. The enrolled COVID-19 patients presented with common symptoms such as fever, cough, and dyspnea at the time of hospital admission. Additionally, as defined by a neurologist, patients were stratified based on the presence or absence of neurological symptoms, such as confusion, syncope, headache, paresthesia, and mental fog.

### 3.2. MiRNA Expression

**Figure 1:**
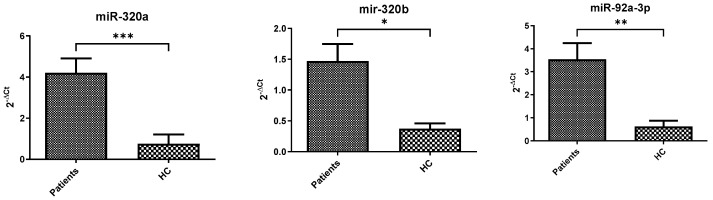
miRNA expression levels (2 − ΔCt) in healthy controls and patients. Y-axis shows relative expression (2^−ΔCt) normalized to miR-191-5p. Data are represented as mean ± standard error; sample sizes for each subgroup (patients and HC) are reported in Table 1 (*): *p* ≤ 0.05, (**): *p* ≤ 0.01, (***): *p* ≤ 0.001.

Figure 1 shows the values of the different concentrations obtained for each group, represented as the difference in means (Δ) ± the standard error of the means (SEM).

**Figure 2:**
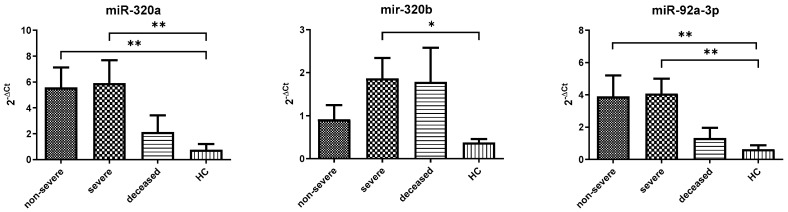
miRNA expression levels among severe and non-severe groups, deceased patients and HC. Y-axis shows relative expression (2^−ΔCt) normalized to miR-191-5p. Data are represented as mean ± standard error. Non-severe patients: VMK and AA; severe patients: IOT and NIV. Sample sizes for each subgroup (IOT, NIV, VMK, and AA) are reported in Table 2; (*): *p* ≤ 0.05, (**): *p* ≤ 0.01.

**Figure 3:**
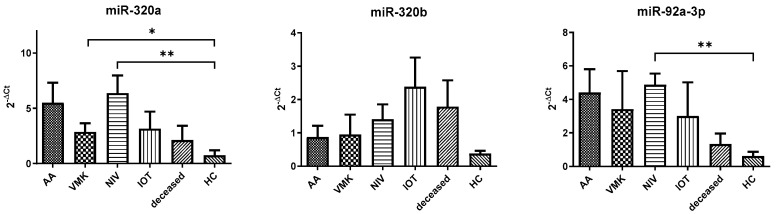
miRNA expression levels (2 − ΔCt) between orotracheal intubation (IOT), noninvasive ventilation (NIV), Venturi mask for oxygen (VMK), room air (AA), deceased and health patients (HCs). Y-axis shows relative expression (2^−ΔCt) normalized to miR-191-5p. Data are represented as mean ± standard error. Sample sizes for each subgroup (IOT, NIV, VMK, and AA) are reported in Table 2. (*): *p* ≤ 0.05, (**): *p* ≤ 0.01.

Compared to healthy controls, COVID-19 patients exhibited a significant increase in all three miRNAs analyzed (Figure 1). miR-320a and miR-92a showed more than fivefold elevation, while miR-320b increased approximately fourfold (mir-320a: 4.20 ± 0.70 vs. 0.75 ± 0.45 *p* = 0.0002; mir-320b: 1.47 ± 0.28 vs. 0.37 ± 0.08 *p* = 0.0118; and mir-92a: 3.55 ± 0.69 vs. 0.63 ± 0.25 *p* = 0.0020). These findings indicate a robust dysregulation of tau-associated miRNAs in COVID-19 patients compared to controls. According to the stratification by severity, miR-320a and miR-92a were significantly increased in both non-severe and severe groups in comparison to HC (Figure 2) (miR-320a: 5.59 ± 1.53 and 5.91 ± 1.79 vs. 0.75 ± 0.45 *p* = 0.0013; miR-92a: 3.91 ± 0.59 *p* = 0.048 and 4.06 ± 0.93 vs. 0.63 ± 0.25 *p* = 0.0086), while miR-320b showed a significant increase only in the severe group (0.91 ± 0.33 vs. 0.37 ± 0.08 *p* = 0.017), suggesting a potential role in disease progression. Further stratification according to ventilation requirements during hospitalization revealed that miR-320a was significantly increased in NIV and VMK groups compared to controls, while miR-92a showed a similar pattern for NIV. miR-320b did not reach statistical significance across subgroups, although a trend toward higher levels was observed in IOT patients (Figure 3). When adjusted using the Benjamini–Hochberg procedure, all significant comparisons remained significant with the exception of the VMK vs. HC comparison for miR-320b in Figure 3, which did not retain significance after correction.

### 3.3. Correlation Analysis

**Figure 4:**
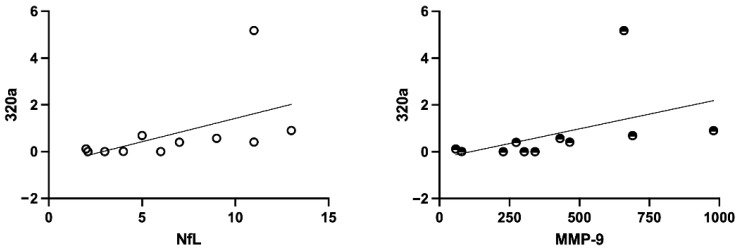
Graphs illustrating Spearman’s correlation analysis in healthy individuals, between miRNA 320a and NfL (rho = 0.756, *p* = 0.007) and between 320a and MMP-9 (rho = 0.745, *p* = 0.012) circulating levels.

We assessed potential correlations between plasma levels of the three tau-associated miRNAs (miR-92a-3p, miR-320a, and miR-320b) and previously published biomarkers of neuroinflammation and neurodegeneration [17,27,28] including MMP-9, MMP-2, TIMP-1, MMP-9/TIMP-1 ratio, sCD14, sCD163, NfL, and GFAP. Among the three miRNAs, only miR-320a showed a positive correlation with NfL and MMP-9 in the health control group (Figure 4). However, this association was lost in COVID-19 patients, regardless of disease severity or ventilation requirements. No significant correlations were observed for miR-92a or miR-320b with any of the biomarkers analyzed. Similarly, all other potential associations between miRNAs and inflammatory or neurodegenerative markers were not statistically significant (all *p* > 0.05). These findings suggest that the regulatory relationship between miR-320a and markers of axonal damage and extracellular matrix remodeling may exist under physiological conditions but becomes disrupted during SARS-CoV-2 infection.

## 4. Discussion

In this single-center retrospective study, we observed that plasma levels of three microRNAs, miR-92a-3p, miR-320a, and miR-320b, were significantly higher in hospitalized COVID-19 patients compared to HC. These miRNAs are known to regulate MAPT expression, encoding tau protein, a key component in neurodegenerative disorders such as AD and FTD [18,19]. The observed dysregulation aligns with previous reports of altered miRNA profiles in COVID-19 and tau-related pathways [20,21]. When patients were stratified by disease severity, miR-320a and miR-92a-3p exhibited consistent up-regulation across both severe and non-severe groups, whereas miR-320b showed a significant increase only in severe cases. This pattern may suggest a potential link between miR-320b and disease progression, although the small number of patients in the most severe subgroup (IOT) precludes firm conclusions. Further stratification by ventilation requirements revealed similar trends, but given the limited sample size for each group, these findings should be interpreted with caution. These results indicate that while miRNA dysregulation is evident in COVID-19, its relationship with clinical severity remains to be clarified [20,29]. Correlation analyses did not demonstrate significant associations between the three miRNAs and established biomarkers of neuroinflammation or neurodegeneration, such as NfL, GFAP, and MMP-9, in COVID-19 patients. Interestingly, a positive correlation between miR-320a and NfL/MMP-9 was observed in healthy controls but was absent from the patient’s cohort. This difference suggests that SARS-CoV-2 infection may disrupt physiological miRNA–protein regulatory networks. Several mechanisms could contribute to this phenomenon. First, NfL and MMP-9 increase during acute SARS-CoV-2 infection mainly because of systemic inflammation, endothelial injury and tissue damage, processes that can saturate or override the fine post-transcriptional regulation normally mediated by circulating miRNAs [30]. Indeed, NfL rises substantially in severe COVID-19 due to widespread axonal stress rather than regulatory control, and MMP-9 release during cytokine activation derives predominantly from activated neutrophils and vascular dysfunction. Second, SARS-CoV-2 profoundly alters miRNA expression, processing and functional targeting, as shown by studies demonstrating global dysregulation of circulating miRNAs during acute infection and post-acute phases [15,31]. These mechanisms collectively could provide a biological rationale for the loss of correlation in the patient’s cohort. The functional relationship between miR 320a and MMP 9 provides a biologically plausible framework for interpreting our findings. Experimental studies have demonstrated that miR 320a directly targets MMP 9 mRNA, suppressing its translation, whereas inhibition of miR 320a increases MMP 9 protein expression and secretion [22]. Although this mechanistic evidence derives from multiple sclerosis models, it is relevant in the context of COVID-19 because MMP 9 contributes to endothelial activation, extracellular matrix remodeling, and blood–brain barrier permeability, all processes strongly implicated in COVID-19 pathophysiology. The absence of the miR 320a–MMP 9 correlation in our patient cohort could therefore reflect a transient disruption of this regulatory axis during SARS-CoV-2 infection, potentially driven by the overwhelming inflammatory environment characteristic of moderate to severe disease. To our knowledge, no physiological associations between these circulating miRNAs and NfL have been previously reported, which is consistent with the lack of correlations observed in our cohort.

Although these miRNAs have been linked to MAPT regulation in experimental systems, an important limitation of our study is that we did not measure total tau (t tau) or phosphorylated tau (p tau) in the plasma samples available. The independent literature has consistently reported that tau-related biomarkers are altered in COVID-19 and correlate with clinically meaningful outcomes. Increased levels of NfL, GFAP, and p tau 181 have been associated with acute disease severity, in hospital mortality, COVID-19-related encephalopathy, and the development of new cognitive symptoms among hospitalized patients [30,32,33,34]. Moreover, p tau 181 elevations have been described not only during acute infection but also during the post-acute phase, and fluctuations in p tau 181 levels were associated with increased risk of amyloid abnormalities and adverse cognitive trajectories in individuals with neurological post-acute sequelae of COVID-19 [34]. These findings support the possibility that COVID-19 may exacerbate neurodegenerative pathways or unmask pre-existing vulnerabilities, although causality remains unproven.

Recent population-based proteomic analyses have extended these observations by showing that SARS-CoV-2 infection may be associated with long-term changes in plasma biomarkers linked to AD risk. A study in UK Biobank participants reported that individuals with prior SARS-CoV-2 infection exhibited biomarker alterations suggestive of higher β amyloid pathology, increased AD-related imaging measures, reduced plasma Aβ42, and elevated p tau 181, particularly among those with higher susceptibility [35]. These findings do not establish a direct causal link but indicate that SARS-CoV-2 and possibly other systemic inflammatory conditions may modulate neurodegenerative pathways over time.

Neuropathological evidence further strengthens this view. Post mortem studies have shown Alzheimer like tau hyperphosphorylation and pronounced neuroinflammation in the hippocampus and entorhinal cortex of individuals who recovered from COVID-19 months before death, suggesting a persistent impact of SARS-CoV-2 on brain homeostasis [10]. Taken together, these data provide a conceptual framework for interpreting the biological relevance of the miRNAs evaluated in our study, while highlighting that any connections between miRNA dysregulation and tau pathology in our cohort must be regarded as hypothetical and exclusively grounded in the external literature, given the absence of direct tau measurements.

This study has several limitations. The sample size was modest, especially when subdividing patients by ventilation categories, which restricts the statistical power of subgroup analyses. The cross-sectional design does not allow assessment of temporal dynamics or causal relationships. Most importantly, although these miRNAs have been implicated in tau-related pathways in experimental systems, we did not quantify tau biomarkers (t-tau or p-tau) in this cohort. This precludes any direct inference about tau pathology and requires caution when interpreting potential mechanistic links. Furthermore, we cannot exclude confounding effects from comorbidities, pre-existing neurological conditions, or therapeutic interventions administered during hospitalization. In our cohort, plasma total tau and phosphorylated tau could not be measured due to insufficient sample volume. These samples were collected during the very first COVID-19 wave, under emergency clinical conditions, and represent a unique and irreplaceable biobank. As such, the available plasma was necessarily limited and fully consumed for the validated biomarker assays included in the present study. The historical and non-reproducible nature of these specimens underscores their scientific value but also imposes constraints on the number of analytes that can be assessed. Taken together, these aspects confirm that our results should be interpreted as descriptive and hypothesis-generating and should be considered exploratory, requiring validation in larger and prospectively designed cohorts. Despite these limitations, our results offer preliminary evidence that tau-associated miRNAs are dysregulated in COVID-19 and may warrant further investigation as potential biomarkers. Although miR 92a 3p and miR 320 family members are not specific to COVID-19 and can be modulated in other inflammatory conditions, several independent studies have consistently reported their up-regulation in SARS-CoV-2 infection, with potential associations to disease severity and outcomes [20,21]. Their known roles in endothelial activation and extracellular matrix remodeling further support their relevance to COVID-19-related microvascular injury, suggesting that their dysregulation in our cohort may reflect neurovascular and inflammatory responses specific to SARS-CoV-2 infection rather than a generic ARDS-related or cytokine-driven signature.

Future studies should adopt longitudinal designs to evaluate whether changes in miR-92a-3p, miR-320a and miR-320b precede or follow alterations in neuronal or astroglia biomarkers. Integrating circulating miRNAs with parallel measurements of tau, p-tau, NfL, GFAP, cytokines and BBB integrity markers will be essential to elucidate their mechanistic significance. In addition, single-cell or neuron-derived extracellular vesicle profiling may help reveal tissue-specific origins of these miRNAs, as recent studies have demonstrated that miR-92a and miR-320a are enriched in neuron-derived vesicles in tau-related neurodegenerative conditions [19]. Finally, multimodal studies combining miRNA signatures with neuroimaging and cognitive follow-up could help determine whether these circulating markers contribute to predicting long-term NeuroCOVID outcomes.

## 5. Conclusions

Despite significant improvements, progress is needed in exploring SARS-CoV-2-related microRNAs. Initially, most of the evidence gathered comes from computational estimates or laboratory experiments, with limited in vivo validation and clinical translation. Subsequently, the precise contribution of various miRNAs in different organs during the multiple phases of both viral infection and subsequent recovery is still not fully understood. Furthermore, individual differences in miRNA expression, determined by factors such as age, gender, previous medical conditions, treatments and circulating viral strains, make the development of reliable biological markers challenging. Finally, challenges related to the delivery of these RNAs, adverse reactions, and long-term health protection continue to hinder the clinical use of miRNA-based treatments. Overcoming these obstacles through long-term research, conducted with rigorous experimental designs and controlled clinical trials, will be essential to fully uncover the therapeutic and diagnostic capabilities of miRNAs in the context of COVID-19 and other viral diseases. In conclusion, while our findings suggest a possible link between SARS-CoV-2 infection and altered regulation of tau-related miRNAs, these observations remain exploratory. Robust prospective studies are needed to confirm these associations and clarify their clinical significance.

## Figures and Tables

**Table 1:** Clinical characteristics of the study population. Summary of demographic features of hospitalized COVID-19 patients and healthy controls included in the study.

Groups	Number	Sex (M/F)	Mean Age ± SD
COVID-19	38	20/18	67 ± 14
Healthy controls	12	6/6	60 ± 9

**Table 2:** Ventilation-based stratification of COVID-19 patients. Distribution of COVID-19 patients according to the maximum level of respiratory support required during hospitalization. IOT and NIV: severe patients; VMK and AA: non-severe patients.

Subgroups	Number	Sex (M/F)	Mean Age ± SD
IOT	4	4/0	71 ± 13
NIV	16	9/7	68 ± 14
VMK	6	2/4	69 ± 14
AA	12	5/7	68 ± 14

## Data Availability

The original contributions presented in this study are included in the article/Supplementary Materials. Further inquiries can be directed to the corresponding authors.

## Supplementary Materials

The following supporting information can be downloaded at: https://www.mdpi.com/article/10.3390/cells15060503/s1.

## Abbreviations

The following abbreviations are used in this manuscript:

AD	Alzheimer Disease
ARDS	Acute Respiratory Distress Syndrome
CT	Computed Tomography
FTD	Frontotemporal Dementia
HC	Healthy Controls
IOT	Orotracheal Intubation
miRNAs	microRNAs
NDs	Neurological Disorders
NfL	Neurofilament Light Chain
NIV	Non-Invasive Ventilation
RT-PCR	Reverse Transcription Polymerase Chain Reaction
VMK	Venturi Mask
