# Supplementary material for: Profile of Tau-Associated Selected MicroRNAs in Hospitalized COVID-19 Patients: An Exploratory Single-Center Study
Source: Cells. 2026 Mar 12;15(6):503. doi: 10.3390/cells15060503 (PMC13025435; doi:10.3390/cells15060503)
Supplement: Supplementary file 1 [file cells-15-00503-s001.zip › cells-4174450-supplementary.pdf]

Power Analysis

For the calculation of the sample size in a pilot study, we relied on Power Analysis, setting acceptable conditions of a 1% Type I error and a test power of 95% (beta error= 10%). The tests were conducted on independent groups using a two-tailed approach to increase the reliability of the test itself. To reduce sample variance, the two populations: healthy controls and patients were matched by sex, age, and other potentially confounding factors. As the starting variable for the power analysis, we selected the plasma levels of miR-320b, as we expected the smallest difference between the two groups, i.e., the smallest delta, according to a previous study that investigated the plasma levels of miR-320b in COVID patients [1] (https://doi.org/10.1038/s41598-024-64325-9).

We expected to observe a miR-320b expression levels (2-ΔCt) of approximately $0,6x10^3$ in controls, with a standard deviation of about $0,5\ x10^3$, whereas COVID patients a a miR-320b expression levels of around $3,4\ x10^4$, with a standard deviation of about $2\ x10^4$.

Below is the calculation report from G*Power:

t tests - Means: Difference between two independent means (two groups)

Analysis: A priori: Compute required sample size

**Input:**	Tail(s)	=	Two
	Effect size d	=	1,920784
	α err prob	=	0,01
	Power (1-β err prob)	=	0,95
	Allocation ratio N2/N1	=	3
**Output:**	Non centrality parameter δ	=	4,6523699
	Critical t	=	2,7632625
	Df	=	28
	Sample size group 1	=	8
	Sample size group 2	=	22
	Total sample size	=	30
	Actual power	=	0,9636007

Reference

[1] de Souza Nicoletti, A., Berlofa Visacri, M., Regina da Silva Correa da Ronda, C. *et al.* Increased expression of miR-320b in blood plasma of patients in response to SARS-CoV-2 infection. *Sci Rep* **14**, 13702 (2024). https://doi.org/10.1038/s41598-024-64325-9
